# Challenges arising when seeking broad consent for health research data sharing: a qualitative study of perspectives in Thailand

**DOI:** 10.1186/s12910-018-0326-x

**Published:** 2018-11-07

**Authors:** Phaik Yeong Cheah, Nattapat Jatupornpimol, Borimas Hanboonkunupakarn, Napat Khirikoekkong, Podjanee Jittamala, Sasithon Pukrittayakamee, Nicholas P. J. Day, Michael Parker, Susan Bull

**Affiliations:** 1 Mahidol Oxford Tropical Medicine Research Unit (MORU), Faculty of Tropical Medicine, Mahidol University, Bangkok, Thailand; 2 Centre for Tropical Medicine and Global Health, Nuffield Department of Clinical Medicine, University of Oxford, Old Road Campus, Roosevelt Drive, Oxford, OX3 7FZ UK; 3 The Ethox Centre, Nuffield Department of Population Health, University of Oxford, Oxford, UK; 4 Department of Clinical Tropical Medicine, Faculty of Tropical Medicine, Mahidol University, Bangkok, Thailand; 5 Shoklo Malaria Research Unit, Faculty of Tropical Medicine, Mahidol University, Bangkok, Thailand; 6 Department of Tropical Hygiene, Faculty of Tropical Medicine, Mahidol University, Bangkok, Thailand; 7 Wellcome Centre for Ethics and Humanities, Nuffield Department of Population Health, University of Oxford, Oxford, UK

**Keywords:** Data sharing, Secondary use, Broad consent, Thailand, Research ethics, Research

## Abstract

**Background:**

Research funders, regulatory agencies, and journals are increasingly expecting that individual-level data from health research will be shared. Broad consent to such sharing is considered appropriate, feasible and acceptable in low- and middle-income settings, but to date limited empirical research has been conducted to inform the design of such processes. We examined stakeholder perspectives about how best to seek broad consent to sharing data from the Mahidol Oxford Tropical Medicine Research Unit, which implemented a data sharing policy and broad consent to data sharing in January 2016.

**Methods:**

Between February and August 2017 qualitative data were collected at two sites, Bangkok and the Thai-Myanmar border town of Mae Sot. We conducted eighteen semi-structured interviews. We also conducted four focus group discussions with a total of nineteen people. Descriptive and thematic coding informed analysis of aspects of data sharing that are considered most important to inform participants about, and the best ways to explain complex and abstract topics relating to data sharing.

**Results:**

The findings demonstrated that clinical trial participants prioritise information about the potential benefits and harms of data sharing. Stakeholders made multiple suggestions for clarifying information provided about data sharing on such topics. There was significant variation amongst stakeholders’ perspectives about how much information should be provided about data sharing, and it was clear that effective information provision should be responsive to the study, the study population, the individual research participant and the research context.

**Conclusions:**

Effectively communicating about data sharing with research participants is challenging in practice, highlighting the importance of robust and effective data sharing governance in this context. Broad consent should incorporate effective and efficient explanations of data sharing to promote informed decision-making, without impeding research participants’ understandings of key aspects of the research from which data will be shared. Further work is required to refine both the development of core information about data sharing to be provided to all research participants, and appropriate solutions for context specific-challenges arising when explaining data sharing.

## Background

Research funders, regulatory agencies, and journals are increasingly expecting that individual-level data obtained from health research will be shared more widely [1–4]. Rationales for sharing data include maximising the utility of datasets and improving the rigour and transparency of research, with the ultimate aim of improving health [5, 6]. Arguments have been made that data generated from health research are a public good and that data should be shared with the wider research community with as few restrictions as possible [7].

Notwithstanding the strength of arguments in favour of rapid sharing of data with minimal restrictions, the importance of seeking appropriate consent to such sharing, in combination with appropriate de-identification of individual-level data and other measures to mitigate potential risks to research participants, has been widely recognized [5, 8]. There is, however, on-going debate about best practices in seeking consent to data sharing, including the merits and challenges of varying approaches, such as broad consent and dynamic consent [9–12].

The 2016 Council for International Organizations of Medical Sciences (CIOMS) guidance on research ethics concludes that it is acceptable for researchers to seek broad consent for unspecified future use of individual patient data [8]. Our previous research in Thailand demonstrated that broad consent has been considered appropriate in our context, provided participant safeguards have been established, echoing findings from other low- and middle- income settings (LMICs) [13, 14]. However, to date there have been few empirical investigations into best practices in seeking broad consent for data sharing in LMICs, including considerations of how much information to provide to research participants and how best to explain concepts comprehensibly. Our experience, and the experience of others conducting research in LMICs shows that research participants often do not comprehend some aspects of research, including abstract and unfamiliar concepts [15–18].

Providing information about data sharing and obtaining broad consent for future unspecified uses of data, in addition to consent to the primary biomedical study from which data will be shared, adds a layer of complexity to the consent process. In order to provide an evidence base for comparison of the merits of different approaches to seeking consent we sought to address these questions by investigating what aspects of data sharing are considered most important to inform research participants about and views about best ways of explaining complex and abstract topics related to data sharing.

## Methods

### Setting

The Mahidol Oxford Tropical Medicine Research Unit (MORU) was established in 1979 as a research collaboration focusing on tropical medicine between Mahidol University in Thailand and the Nuffield Department of Medicine, University of Oxford in the United Kingdom. The main office and laboratories are located within the Faculty of Tropical Medicine in Bangkok, Thailand, but research is conducted in many different locations both in Southeast Asia and more widely in South Asia and Africa, where the disease burden of tropical diseases are high. At any one time, MORU coordinates around 60 to 70 active clinical studies on malaria and other neglected diseases such as melioidosis and unexplained fevers. The studies range from small single-centre studies to large multicentre studies recruiting tens of thousands of participants. In recent years, MORU has coordinated some of the largest international studies involving many sites in low-income and hard-to-reach settings in tropical diseases such as malaria [19–21]. The majority of the studies coordinated by MORU are sponsored by the University of Oxford and funded by charities such as the UK’s Wellcome Trust and the Bill and Melinda Gates Foundation. In such studies, Bangkok, with the support of the in-house Clinical Trials Support Group, has acted as the hub for study management, coordination and data management. With more than 800 personnel distributed across its collaborative research network, MORU and its partners generate vast amounts of research data every year.

It has been MORU’s policy for many years to support sharing of data across collaborative research networks and more widely to maximize its research impact. In order to formalize the process of data sharing, in January 2016, MORU established a data sharing policy in which requests for data are channeled through a Data Access Committee (DAC) and discussed with the senior investigators of the relevant studies [22–24]. The MORU data sharing policy was informed by a collaborative study into best practices in sharing individual level data generated in LMICs in 2014–2015 [13, 14]. A series of internal consultations with MORU scientists and a review of our main funders’ policies as well as those of leading journals also informed the policy development.

In the 2014–2015 collaborative data sharing study, interviews and focus group discussions were conducted in Kenya, South Africa, Vietnam, India and Thailand with stakeholders including researchers, community representatives and research participants [13, 25–29]. Participants in this study recognised both benefits and potential harms of sharing individual-level data. At MORU, it was felt that the best way to promote potential benefits and ameliorate potential harms to data subjects, primary researchers, collaborators, and to promote public trust, would be through the adoption of a managed access approach with broad consent to research being obtained from research participants [13].

### Study sites and interviewees

This qualitative study was conducted at two sites. The first site was the MORU-affiliated healthy volunteer ward in Bangkok where healthy volunteer phase I and pharmacokinetic studies are routinely conducted. These studies often recruit young and middle-aged Thai adults, some of whom are students and staff from nearby universities and hospitals. This site was chosen as it has a large pool of individuals best placed to understand data sharing and provide broad consent to data sharing, and who are generally healthy, educated and able to participate in semi-structured interviews (SSIs) and focus group discussions (FGDs). At this site, we conducted SSIs and FGDs with clinical trial participants, research interns and researchers. The majority of clinical trial participants have completed high school, while research interns were undergraduate students, and researchers had bachelor or postgraduate degrees. We also conducted an FGD with an ad hoc “Health Research Interest Group” comprising of Thai and other Asian postgraduate students from nearby universities and others interested in health research.

The second site was MORU’s biggest research site, the Shoklo Malaria Research Unit (SMRU), which has a hub at the Thai–Myanmar border town of Mae Sot. SMRU has been involved in providing health care and conducting research with the Burmese and Karen migrant population on the Thai–Myanmar border zones for more than 30 years. The focus of research has been on infectious diseases such as malaria, and maternal and child health, the main healthcare burdens of the border population. There is limited access to medical personnel and facilities on either side of the border, hence many migrants access SMRU and non-governmental organization clinics. In contrast to the Bangkok site, the population in Mae Sot are relatively poor and many of them are illiterate. At this site, we conducted SSIs with researchers and an FGD with the long standing Tak Province Community Ethics Advisory Board (CAB) [30, 31]. All researchers had either bachelor or postgraduate degrees. The CAB consists of ethnic Karen and Burmese individuals who reside along the Thai-Myanmar border, majority have completed primary education. Due to the abstract nature of the subject we chose not to include clinical research participants in Mae Sot at this stage. We anticipated that the current study would give us insight on how we could better explain data sharing to participants in order to maximize their understanding, which could inform future research into their opinions about data sharing.

### Data collection and analysis

As our objective was to gather experiences and views from a range of relevant stakeholders, we used a combination of purposive and convenience sampling. We recruited three groups of participants: 1) clinical trial participants recruited into healthy volunteer studies who had consented to data sharing, 2) researchers directly involved in seeking broad consent from participants, those involved in the implementation of the MORU data sharing policy and research interns, and 3) community members with an interest in health research i.e. the Tak Province Community Ethics Advisory Board and the Bangkok Health Research Interest Group. None of the interviewees approached for SSIs refused to be interviewed. For FGDs, we invited potential interviewees by email or in person to attend the FGD at a specific time and place.

Between February and August 2017, we conducted eighteen SSIs. We also conducted four FGDs with a total of nineteen participants (Table 1). We also reviewed minutes from internal MORU meetings where data sharing was discussed.

**Table 1:** Numbers of semi-structured interviews and focus group discussions in Bangkok and Mae Sot

	Semi-structured interviews	Focus group discussions
Bangkok	13	3
Mae Sot	5	1
Total	18	4

SSIs and FGDs were conducted using topic guides and were in the preferred language of the participants (English, Thai or Karen). PYC, BH, NJ and SB developed separate topic guides for each group, which were responsive to group members’ prior experience with data sharing. SSIs and FGDs were conducted by PYC, NJ, NK and BH.

**Fig. 1:**
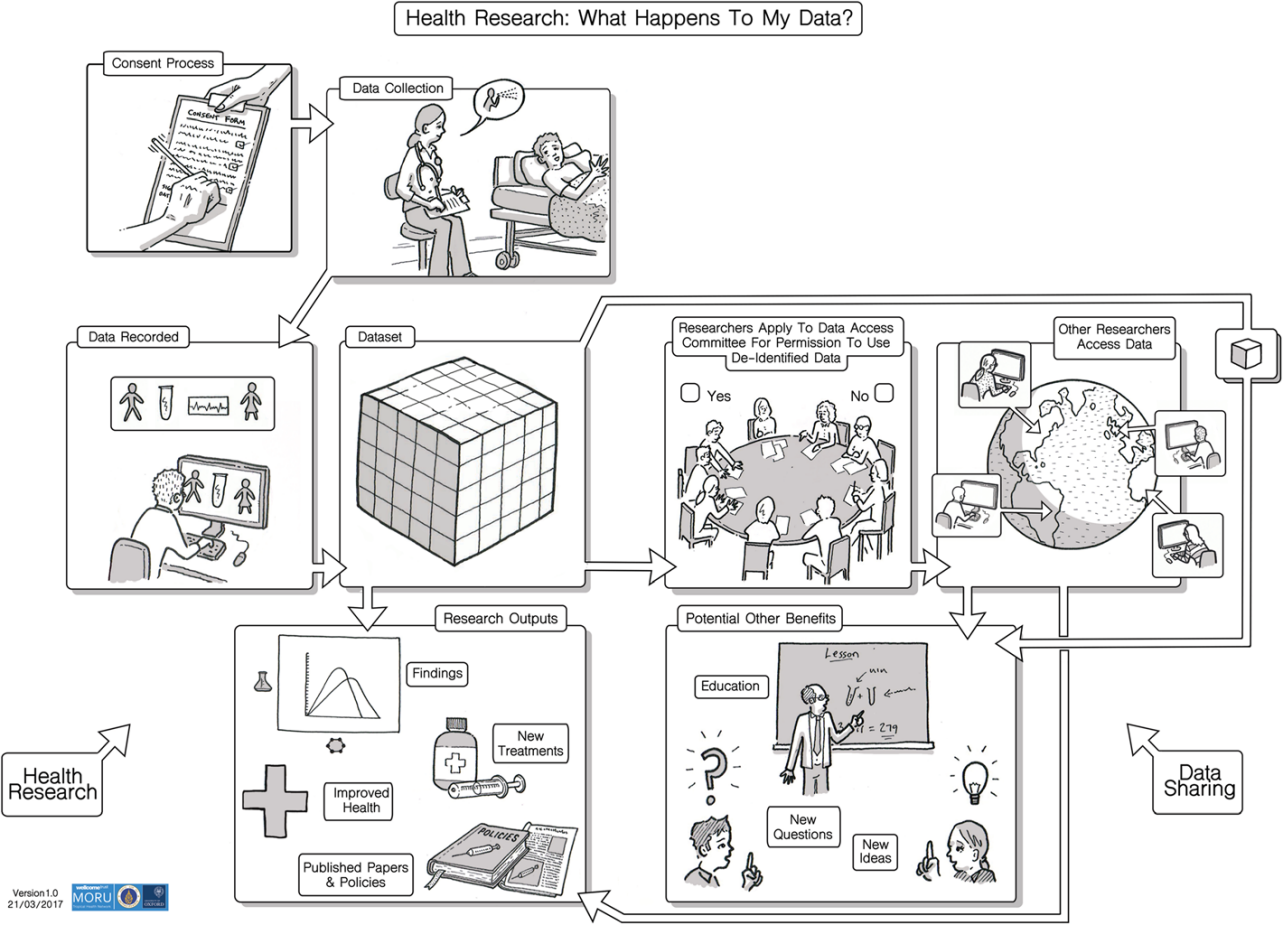
Data sharing illustration

We encountered many challenges when explaining data sharing. In order to support introductory explanations of data sharing during SSIs and FGDs, we developed and refined a pictorial representation of data sharing with the help of a professional illustrator (Fig. 1). The illustration was designed to show what happens to data in health research, and when de-identified individual level research data are shared via the MORU Data Access Committee.

During the research, PYC, BH, SB, and NJ met to review initial transcripts and revise the guides to aid clarity and flow, as well as to probe information gaps and emerging themes. All SSIs and FGDs started with a brief introduction to data sharing and a short description of the MORU data sharing policy, including the data governance process. [22] The topic guides focused on exploring current understandings of MORU data sharing practices; views about what information it is most important to provide about data sharing during recruitment; how best to explain complex and abstract aspects of data sharing. All SSIs and FGDs were conducted at a time and place convenient to interviewees, audio recorded, transcribed and translated to English where necessary.

The data analysis was informed by framework analysis approaches as the study addressed predefined questions informed by prior research and practice, and sought to inform actionable outcomes (refinements to consent processes for healthcare research where data sharing is anticipated) [14, 32]. PYC, NJ and SB reviewed all transcripts to familiarize themselves with the richness and diversity of the data. Transcripts were then imported into Nvivo 10 for coding and analysis. Initial descriptive coding was conducted by NJ and PYC, exploring the views of participants. Transcripts were subsequently independently coded by SB, and the initial coding was refined after discussion of the themes emerging from the data.

All participants signed a consent form in their preferred language prior to participation. They were specifically asked if they would consent for their de-identified data to be shared via MORU’s managed access route and all agreed.

### Ethics approvals

Approval for study was obtained from the Oxford Tropical Research Ethics Committee (OxTREC Ref.: 5119–16) and from the Faculty of Tropical Medicine Ethics Committee, Mahidol University (FTM EC Ref.: TMEC 16–101).

## Results

Clinical trial participants had clear views about priority topics they wanted information about when broad consent to data sharing is sought. However, views about how much data should be provided to research participants, and how best to promote understanding, varied substantially, and were informed by the contexts from which interviewees were drawn. These are discussed in turn below. The results are presented according to four themes. In each theme, we start with perspectives from clinical trial participants, followed by those from researchers and community members.

### What is it important to know about data sharing?

The majority of clinical trial participants interviewed were of the opinion that as long as data sharing had potential benefits to society and they would not be harmed or inconvenienced by such sharing then they were happy to consent to it.

*‘If it could benefit others in the future I’m OK with sharing data to others.’ (P7, clinical trial participant, female, SSI, Bangkok).*

*‘It’s the data that is already collected, so it think it’s OK to share it. It’s something that doesn’t have a negative impact to me anyway.’ (P6, clinical trial participant, male, SSI, Bangkok).*

More specifically, the most commonly occurring topics arising in questions clinical trial participants asked during interviews and their responses to interview prompts, suggest that there are four priority areas that they would like to receive information about when consenting to data sharing. These are: that sharing research data has potential benefits; that data will be de-identified; that no foreseeable individual or group harms will come to the research participants; and that there will not be any foreseeable additional inconvenience or burdens incurred by agreeing to data sharing.

When interviewed, the majority of clinical trial participants spontaneously sought to confirm if data would be de-identified before sharing. When they were asked if they were willing to allow their data to be shared more widely, all agreed, provided that they could not be identified. Many also implied that they knew that de-identification was standard in clinical trials, which was unsurprising given that they are familiar with the activities of the MORU-affiliated health volunteer ward, and de-identification of samples and data is routinely discussed during trial recruitment.

*‘Yes, I am willing to share that but only blood result, not my name, my first name. The rest are fine, including blood sample or blood test result.’ (P2, clinical trial participant, male, SSI, Bangkok).*

*‘She asked whether I am willing to participate or is it okay for my data to be shared. She told me that the data that will be shared contains no names or any of my identification. So, I told her it is ok. I will give my consent.’ (P3, clinical trial participant, female, SSI, Bangkok*).

In addition to ensuring that names were kept confidential, some clinical trial participants noted that they would not want their location, addresses and phone numbers to be shared. In this context, where healthy volunteers were taking part in pharmacokinetic research, participants did not express concerns about potential harms of being identified as taking part in a specific study. Instead, they expressed concerns about their identifiable information being used by third parties, such as telemarketers and insurance agencies, for purposes unrelated to health research.

Clinical trial participants also raised concerns about potential additional responsibilities arising from consenting to data sharing. For example, a participant asked, *‘So the consent to data sharing in this sense means that I have to come back here again or just only this time? (P7, clinical trial participant, female, SSI, Bangkok).* She mentioned that she did not mind being re-contacted by phone but she would be reluctant to attend extra meetings about data sharing as she would not want to take leave from her work. This suggests that research participants should be informed while consenting to data sharing that such sharing will not result in any additional burdens to them and how any possible recontact will be managed.

In contrast, researchers and community members noted the importance of de-identification in minimising harms to research participants, and raised concerns that de-identification at the individual level is not adequate to prevent community or group-based harms. They explained that this was because some groups, such as migrant workers on the Thai-Myanmar border, may face increased stigmatization if they are identified as being a source of infectious diseases like malaria. To mitigate these ethical challenges, one researcher suggested that it is necessary for participants to be assured in the consent process that data would be shared in a way that would minimize any harm to them and their communities *(R6, researcher, male, SSI, Mae Sot).*

The following sections review perspectives about how much information should be provided on these priority topics and how best to explain complex aspects of data sharing.

### How much information should be provided about data sharing?

Clinical trial participants did not have strong opinions about how much information about data sharing should be provided.

However, there was substantial variation in researchers and community members’ views about how much information should be provided about data sharing when broad consent was sought. Some believed that detailed information should be given, while others thought that it was too burdensome to explain data sharing in detail.

Some researchers commented on the importance of tailoring consent to context, and suggested that as with the primary healthcare study, the amount of information to be provided about data sharing should be responsive to research participants’ health condition and state of mind at the time of enrolment in a biomedical study, their interest, level of literacy, background knowledge about research and their culture. When research participants are unwell at the time of recruitment, a two-stage process may be appropriate with specific consent for the primary study being sought at recruitment, with broad consent being sought when the patient has recovered enough to understand the details of data sharing.

*‘It depends on, I think, it may be because of the clinical condition and also the another thing is their education level…we have to make a balance in our ill patients so that sometime the patient they just understand only main information, the objective of the study if they are going to involve the benefit and the advantage of the study, they know only those things. And then when they are getting better we have to explain again what we are taking. For example, like after a week of the study and then we have to explain again so at that time they feel much better and then they understand.’ (R8, researcher, female, SSI, Mae Sot).*

In the quotes below a long serving CAB member stressed the importance of a more detailed explanation, while a participant from the Health Research Interest Group suggested that it was not necessary to go into too much detail about data sharing as that could be confusing and alarming for research participants.

*‘We have to take time to explain until they (the clinical trial participants) understand. The bulls will not pull the cart and take off right after you connect the cart to them. As for us, we have been committee members for many years so once you explain, we understand immediately. New people will not understand it…Carefully explain one by one step by step.’ (CAB2, CAB member, male, FGD, Mae Sot).*

*‘…only difference between these two (without data sharing and with data sharing) is one will be used one time, that’s it…. that the data will be used by several different departments or different groups and isn’t that enough?*’ *(HRIG2, HRIG member, female, FGD, Bangkok).*

### Understandings of data sharing

We asked clinical trial participants about whether and how data sharing was explained during the consent process for the clinical trial. They said that they had been provided with enough information, but we found that some did not clearly understand data sharing or had difficulty recalling the information provided about data sharing. This finding is congruent with an earlier empirical study on understandings of consent in our setting, where many respondents commented that they could not remember or understand everything that was explained to them about research [15]. We also found that the words “data”, “sharing” and “data sharing”, as we understand them in this context, and as they were translated into Thai, were not immediately understood by the clinical trial participants, and as a consequence they exhibited a wide range of interpretations of data sharing.

It also became apparent that some researchers and community members did not feel fully confident that they understood the nuances of data sharing. This could be because they had not been involved in data sharing decisions for studies from which data was requested by secondary users. The MORU data governance process involves a review by the DAC in consultation with only senior investigators of the studies from which data are being shared. Field workers, junior staff and CAB members may not have been fully aware of the data sharing policy or the governance process. Consequently some researchers also assumed that data sharing was always via open access mechanisms, meaning that shared research data would be available online for anyone to use. Many researchers were familiar with open access as they had made their data available online as supplementary files to a journal article. Others had uploaded their datasets onto external repositories where datasets can be accessed by registered users who have agreed to the repository’s terms and conditions of use. A contrasting assumption was that data are only shared with collaborators working on the same topic: *‘there may be other researchers in (research unit) or outside (research unit) who are interested in the same topic and so we may share that health information with them’ (R9, researcher, female, SSI, Mae Sot).* The importance of ensuring that researchers have a clear understanding of institutional policies and processes for data sharing, so that these can be accurately discussed with research participants, is considered below in the discussion section.

### Suggestions for promoting understanding

Due to the varying understandings of data sharing, interviewees were prompted to describe such sharing in their own words. When seeking to describe data sharing, clinical trial participants from Bangkok, where the majority of the population are familiar with the internet and social media, drew analogies and likened data sharing to sharing information on social media: *‘Share can be many things like sharing on the Internet etc.’ (P3, clinical trial participant, female, SSI, Bangkok)*. However, others recognized ways in which privacy was protected during research data sharing and described it as the opposite of Facebook, *‘It’s like the opposite...you can know somebody’s name but you know nothing else until you are friends with them.’ (I2, research intern, female, FGD, Bangkok).* This could be because the word for “data” in Thai, “khor moon” is the same word as “information”, and that one common usage of the word “share” refers to hitting the share button to openly share information on social media.

Researchers in the healthy volunteer ward in Bangkok thought that it could be helpful to use the illustration when consenting clinical trial participants and have it displayed in poster format on the walls of the waiting area of the healthy volunteer ward. In contrast, respondents in Mae Sot had mixed feelings about the value of the illustration, saying that it might be too complicated for patients with less education. One researcher explained that most of her patients are illiterate and are not familiar with the concept of clinical research and said *‘I am a hundred percent sure that ninety-nine percent of our patients would be totally bewildered by this [illustration]’ (R9, researcher, female, SSI, Mae Sot).* CAB members in Mae Sot were in favour of using the illustration, saying that *‘you need to spend more time to explain that and then using a tool like visualize or photos is better because it is easier to understand than text’ (CAB2, CAB member, male, FGD, Mae Sot).*

Given that data sharing is difficult to explain, we also asked respondents for suggestions about how to improve the wording about it in the information sheet and consent form. The information sheet for this study contained typical language used in MORU studies, approved by the Faculty of Tropical Medicine Ethics Committee, Mahidol University. During consent processes, this information is supplemented by verbal information. The information sheet stated *“De-identified data from this study may be shared with other groups of researchers. All applications for data sharing will be reviewed by a Data Access Committee. All researchers accessing data need to adhere to a set of terms and conditions that aim to protect the interests of research participants and other relevant stakeholders”,* and in the consent form, *“By signing this page, I agree that de-identified data from this study may be shared with other groups of researchers”.*

Some researchers and community members thought that the wording was clear, while others suggested a range of potential improvements. Some recommendations focused on simplifying the language to promote comprehension, for example ‘*data will be used by several different departments or different groups’ (HRIG2, HRIG member, female, FGD, Bangkok).* Other suggestions focused on clarifying and explaining concepts such as de-identification and secondary data users. “De-identification” was considered potentially confusing because the word “identity” is not usually used in daily conversation. One useful way proposed to describe de-identification was *“data without your personal information for example your name, address, phone numbers etc”* (*khor moon thee mai rabu tuaton* in Thai) (N1, researcher, female, FGD, Bangkok). Researchers also discussed the importance of clear descriptions of who the data may be shared with.

*‘…we should explain …we are not the only organization to use the data. So, after taking a biological sample and the patient information, we will share with other countries. You know, it (countries) is quite understandable for the patient, I think. Other organizations or other they don’t know, NGOs or government or…? In our own words, it is called the organization is not very clear, police, army? All are organizations, you know…and in Myanmar we have research centre of course, but they never heard about it.’ (R7, researcher, male, SSI, Mae Sot).*

## Discussion

This study examined perspectives about how best to seek broad consent to data sharing at MORU, which implemented a data sharing policy and the use of broad consent in January 2016 [23, 24]. To date there are very few empirical accounts on the challenges of seeking broad consent in low- and middle-income settings, the majority of previous studies have been conducted in the context of genomic and biobanking research and in high-income settings [14, 33].

Broad consent has been proposed as an appropriate method for potential research participants to give permission for their samples or data to be used in future research studies [8, 34]. Opponents of broad consent argue that consent cannot be sufficiently informed to be valid if one does not know what the data will be used for in the future [35]. Proponents of broad consent argue that valid consent can take different forms, and that given adequate protections, broad consent can be justified by an appeal to the principle of respect for autonomy [9]. The argument is that broad consent is at least in part a decision to allow others (e.g. a data access committee) to decide, and that consent is to a process of governance [36]. In such circumstances it’s important that the governance structure is robust and trustworthy [8, 9]. Previous research in low- and middle-income settings demonstrates that provided appropriate governance has been implemented, populations are generally supportive of broad consent models for data sharing [14]. Contrasting approaches, such as re-contacting participants for specific consent to future uses of data, were perceived as burdensome and posing risks to patient privacy in such contexts. While broad consent to data sharing is considered to be ethical and culturally acceptable in principle in low- and middle-income settings such as Thailand, this study demonstrates that questions remain about how best to seek such consent in practice.

In this study, the clinical trial participants interviewed did not spontaneously ask about the governance process for data sharing. This could imply that as long as participants know that data may be shared with secondary users, and that risks and harms are appropriately managed, participants are not necessarily interested in the details of data sharing such as the functions of the Data Access Committee. Clinical trial participants’ limited recognition of potential harms of sharing de-identified data, and acceptance of the assurance provided during consent processes about management of risks and harms, emphasizes the importance of an effective governance process to protect the interests of participants and their communities. An important question then is what type of governance process is ideal. The CIOMS guidelines suggest that the governance structure must have “representation from the original setting” and that it must be “robust and trustworthy” [8], but how this process is operationalized needs further empirical and conceptual work.

### Recommendations

Based on the findings of our study we set out to provide some recommendations. A core consideration in the design of research processes incorporating consent to data sharing, is to ensure that participants receive clear information on key issues informing their decision about data sharing. Whilst the concept of ‘broad consent’ acknowledges that detailed and specific consent for all future research uses is not feasible, this research identified four key items that respondents thought was important for research participants to understand in order for broad consent to be considered valid: that data sharing has potential benefits, that the data will be de-identified, that mechanisms are in place to minimize potential harms to participants, and that participants will not be inconvenienced or burdened by such sharing. Researchers and community members suggested ways in which the text about data sharing could be modified to explicitly cover each of these topics, in language that is clear and understandable for research participants. In response to these findings, we propose to amend the language on data sharing in future information sheets, and will consult with stakeholders about the following draft revision: *“Your data has potential benefits beyond the present study, so your data may be used many times and by different groups. These groups will not know your personal information, for example your name, address or phone number. A committee will check each time a group asks to use the data to make sure there are no foreseeable harms to you. If your data is shared you do not need to do anything else such as come to the study facilities again to take part in additional interviews.”* These study findings also demonstrated that researchers may themselves have misunderstandings about data sharing. Examples identified in this study included a lack of understanding that datasets are not always open access and that data can be shared with others not working on the “same topic”. Another important practical area to address in this setting is thus to clarifying misunderstandings of data sharing policies and processes amongst staff recruiting participants so that they are not communicated to patients during consent processes.

Clarifying language about key topics of interest to research participants, and ensuring misunderstandings amongst researchers recruiting participants are addressed are both critical to promoting best practices in seeking broad consent to research in this setting. However, the findings reported here demonstrate that while such measures are necessary, they are not sufficient and additional complex issues remain to be addressed about how much information to provide and how best to explain complex and abstract aspects of data sharing to ensure that participants understand what they are giving broad consent to. Respondents’ perspectives’ varied substantially on these topics, ranging from suggestions that minimal additional information should be routinely provided about data sharing, to suggesting substantial time and effort be spent by researchers and participants to promote thorough understanding of data sharing and its implications. In contrast, some researchers stressed the importance of ensuring that information provided about a healthcare study, and consequent data sharing, is appropriately tailored to specific studies and contexts [37].

In practice, data sharing can be challenging to explain, and the data from this study show that even healthy volunteers with relatively high levels of familiarity with research concepts and social media rarely fully understood or recalled it. Findings from this research additionally suggest that explanations of data sharing need to be carefully tailored to research populations. While research has demonstrated that providing information using audiovisual methods can be promote understanding in LMICs, a systematic review suggests that the evidence base for implementing such methods is poor [38–40]. The findings from this study suggest that use of the figure developed during this study, complemented with analogies and comparisons with sharing via social media, can promote understandings of sharing research data in educated and relatively electronically literate populations in Bangkok. In such settings, it is important that information provision is responsive to the ways in which language about core terms such as ‘sharing’ and ‘data’ is evolving in response to increasing internet usage. In contrast, in Mae Sot there was consensus amongst CAB members and researchers that it would take a lot of work and time to explain the nuances of data sharing clearly to a less literate rural population with limited internet access. In this environment, use of the figure was thought to be of limited value without substantial appropriate background information.

When recruiting participants into research, data sharing is yet one more piece of information to add to the twenty elements that Good Clinical Practice (GCP) guidelines stipulate should be provided about a healthcare study [41]. A study we conducted in Bangladesh showed that including all the GCP-required items in the information sheet did not lead to “informed” consent [15]. Empirical research in other LMIC settings also demonstrate that challenges arise when seeking appropriately informed consent [16, 17]. When seeking broad consent to data sharing, it is critical to recognize the potential tension between providing adequate information about data sharing, and ensuring that important information about the healthcare study from which data will be shared is understood. This may be particularly challenging in rural populations, where greater efforts and more time are likely to be needed to effectively explain data sharing. In practice, research participants may not feel that information about data sharing is a priority to engage with and understand, in comparison with the information they receive about potential benefits, harms and burdens of the biomedical study from which data will be shared [42, 43]. In such circumstances, concerns arise that too much information about data sharing may ‘crowd out’ information about the biomedical study from which data are to be shared, and adversely impact participants’ understandings of the study [26]. Further empirical and conceptual research is needed to inform the development of best practices for efficiently and effectively providing appropriate information about data sharing in such circumstances.

### Future research

A limitation of this study is that only clinical trial participants from healthy volunteer studies in Bangkok were interviewed. These participants are more educated and literate about research than the general population, and may have different motivations and understandings than ill participants who present at a clinic for treatment and are offered the opportunity to participate in research. Further research with research participants from a range of settings in a variety of studies would be valuable to inform the development of materials to appropriately inform participants about data sharing. Another area to explore is challenges relating to seeking surrogate consent in research with children. Sharing data from paediatric trials in LMICs is critical because there are a disproportionately smaller number of research studies conducted with children compared to adults, and thus the sharing of datasets from such studies can be particularly valuable in order to promote paediatric health.

In addition, the researchers interviewed for this study were embedded in or collaborating with a relatively well-resourced institution involved primarily in research on tropical medicine and infectious diseases such as malaria. Some infectious diseases are more stigmatized than others; seeking broad consent to data sharing from research into more stigmatized conditions, such as HIV, may raise additional challenges to those discussed in this context. Challenges relating to seeking broad consent in this context may also be different from challenges faced by researchers working in areas including non-communicable diseases, genomics and rare diseases.

## Conclusions

Communicating effectively about data sharing with research participants is challenging in practice, highlighting the importance of robust and effective data sharing governance in this context. Understandings of data sharing and views on how best to provide information about data sharing vary substantially, and in response to context and participant population, emphasizing the importance of ensuring that information provision is appropriately tailored to specific studies and research contexts. A key consideration in the development of all consent processes is the need to develop effective and efficient explanations which promote informed decision-making about data sharing without impeding participants’ understandings of key aspects of the health research from which data will be shared. Further work, informed by stakeholder and community engagement, is required to refine both the development of core information about data sharing to be provided to all research participants, and appropriate solutions for context specific-challenges arising when explaining data sharing.

## Abbreviations

CAB: community advisory board
DAC: Data Access Committee
FGD: focus group discussion
GCP: Good Clinical Practice
LMIC: lower- and middle-income country
MORU: Mahidol Oxford Tropical Medicine Research Unit
SSI: semi-structured interview
